# Developing and validating a nomogram for penile cancer survival: A comprehensive study based on SEER and Chinese data

**DOI:** 10.1002/cam4.7111

**Published:** 2024-04-03

**Authors:** Jiawen Luo, Jintao Hu, Yelisudan Mulati, Zhikai Wu, Cong Lai, Degeng Kong, Cheng Liu, Kewei Xu

**Affiliations:** ^1^ Department of Urology, Sun Yat‐sen Memorial Hospital Sun Yat‐sen University Guangzhou China; ^2^ Guangdong Provincial Key Laboratory of Malignant Tumor Epigenetics and Gene Regulation, Sun Yat‐sen Memorial Hospital Sun Yat‐sen University Guangzhou China; ^3^ Guangdong Provincial Clinical Research Center for Urological Diseases Guangdong China

**Keywords:** external verification, nomogram, overall survival, penile cancer, prognosis, SEER, TMN

## Abstract

**Objective:**

The primary aim of this study was to create a nomogram for predicting survival outcomes in penile cancer patients, utilizing data from the Surveillance, Epidemiology, and End Results (SEER) and a Chinese organization.

**Methods:**

Our study involved a cohort of 5744 patients diagnosed with penile cancer from the SEER database, spanning from 2004 to 2019. In addition, 103 patients with penile cancer from Sun Yat‐sen Memorial Hospital of Sun Yat‐sen University were included during the same period. Based on the results of regression analysis, a nomogram is constructed and validated internally and externally. The predictive performance of the model was evaluated by concordance index (c‐index), area under the curve, decision curve analysis, and calibration curve, in internal and external datasets. Finally, the prediction efficiency is compared with the TNM staging model.

**Results:**

A total of 3154 penile patients were randomly divided into the training group and the internal validation group at a ratio of 2:1. Nine independent risk factors were identified, including age, race, marital status, tumor grade, histology, TNM stage, and the surgical approach. Based on these factors, a nomogram was constructed to predict OS. The nomogram demonstrated relatively better consistency, predictive accuracy, and clinical relevance, with a c‐index over 0.73 (in the training cohort, the validation cohort, and externally validation cohort.) These evaluation indexes are far better than the TNM staging system.

**Conclusion:**

Penile cancer, often overlooked in research, has lacked detailed investigative focus and guidelines. This study stands as the first to validate penile cancer prognosis using extensive data from the SEER database, supplemented by data from our own institution. Our findings equip surgeons with an essential tool to predict the prognosis of penile cancer better suited than TNM, thereby enhancing clinical decision‐making processes.

## 1 INTRODUCTION

Penile carcinoma, while relatively uncommon in the genitourinary system, once had a higher prevalence in certain regions. Globally, approximately 26,000 new cases are estimated to occur, accounting for approximately 1% of new cancer diagnoses in males worldwide.^1^ Postoperative penile cancer patients often face complex challenges, including concerns about survival, physical and psychological health needs, and adjustments in their lifestyle and relationships. In Western industrialized nations such as Europe and the United States, the incidence varies between 0.1 and 1.0 per 100,000 individuals, whereas in Brazil, it can be as high as 2.8 to 6.8 per 100,000.^2^, ^3^ Squamous cell carcinoma accounts for more than 95% of all penile cancer cases.^4^, ^5^ Treatment options for penile cancer encompass various approaches, including local excision with organ preservation, partial penile excision, radical penile excision, and inguinal lymph node dissection. However, it is worth noting that there is a dearth of comprehensive, large‐scale randomized controlled trials or comparative observational studies assessing the efficacy of primary penile cancer treatments.

Prognostic factors in penile carcinoma encompass a range of variables, including pathological characteristics, clinical stage, lymph node metastasis, and molecular factors. Pathological features such as tumor type, grade, depth of invasion, nerve invasion, and lymphatic canal invasion are pivotal prognostic indicators for penile carcinoma. Numerous studies have revealed the predictive role of pathological grade in metastasis, disease progression, and overall prognosis. Nevertheless, certain factors, such as the primary tumor site, vascular infiltration, treatment modality, and socioeconomic variables like social relationships and family income, have been relatively underexplored in terms of their prognostic implications. Zini et al.^6^ developed a straightforward model for predicting the necessity of surgery in primary penile squamous cell carcinoma, incorporating Surveillance, Epidemiology, and End Results (SEER) stage and tumor grade (TG) as key variables. Furthermore, Thuret et al.^7^ reported that augmenting the AJCC stage with TG enhances the accuracy of predicting cancer‐specific mortality.

A multitude of factors collectively influence the prognosis of penile cancer. Prognostic models derived from the comprehensive SEER database have consistently demonstrated robust predictive capabilities for both overall survival (OS) and cancer‐specific survival (CSS) among patients afflicted with penile cancer.^8^ Therefore, the main goal of this study was to create a validated and reliable nomogram specifically designed to predict survival outcomes for individuals diagnosed with penile cancer, drawing upon data sourced from the SEER and a Chinese organization.

## 2 METHODS

### 2.1 Patients and data collection

During the study period spanning from 2004 to 2019, we identified a total of 5744 patients diagnosed with penile cancer in the SEER database by employing SEER*Stat software (version 8.4.1). Additionally, we conducted a retrospective analysis of 103 patients with penile cancer from Sun Yat‐sen Memorial Hospital (2004–2019), serving as an external validation cohort. The study protocol received approval from the Ethics Committee of Sun Yat‐sen Memorial Hospital, Sun Yat‐sen University, and informed consent was waived. The inclusion criteria were as follows: (1) patients ranging in age from 0 to 100 years old; (2) patients with complete follow‐up information; and (3) patients presenting a single primary tumor, with the exclusion of cases involving other tumors that could potentially influence prognosis.

### 2.2 Development and validation of the nomogram

The selected patients were randomly allocated into two distinct groups: the training cohort and validation group 1, maintaining a ratio of 7:3. Additionally, a validation group 2 consisting of 103 patients from Sun Yat‐sen Memorial Hospital was included for external validation. In these three groups (the training cohort, the internal validation group, and the external validation group), we leveraged significant variables identified through both univariate and multivariate Cox regression models to construct nomograms designed for the precise prediction of OS in cases of penile cancer. The predictive accuracy of our nomogram was assessed by receiver operating characteristic (ROC) methodology; the area under the ROC curve (AUC) and the concordance index (c‐index) measured the discrimination ability. Reliability was analyzed by calibration curves. The prediction model was compared with a prediction model built from TNM stages.

### 2.3 Definitions

Marital status was assessed and categorized as follows: married and other/unknown. Income status was defined as an annual income of <$55,000 or >$55,000. The rural–urban classification was delineated as: Nonmetropolitan (nonmetropolitan counties not adjacent to a metropolitan area and unknown/missing/no match); Metropolitan areas are categorized into three groups: counties situated in metropolitan areas with a population exceeding 1 million, counties within metropolitan regions hosting populations ranging from 250,000 to 1 million, and counties located in metropolitan areas with fewer than 250,000 residents. Additionally, there are “Adjacent to metropolitan” regions, encompassing nonmetropolitan counties situated in proximity to metropolitan areas. The training cohort underwent analysis via the Cox regression model, considering 14 potential variables: age, ethnicity, marital status, income, rural or urban residence, primary site, grade, histology, TNM stage, surgery, non‐primary site surgery, radiation, chemotherapy, and L/V invasion.

### 2.4 Statistical analysis

Statistical differences were described in the figure legends. Statistical analysis was performed employing IBM SPSS Statistics 26.0 and R software, specifically version 4.0.3, which can be accessed at http://www.R‐project.org. The mean, often referred to as the average, is a measure of central tendency that summarizes the central value of a dataset. The standard deviation is a measure of the amount of variation or dispersion of a set of values. The 95% confidence interval (95% CI) is used to estimate the range within which we expect the true value of a population parameter (like the mean or proportion) to lie, with a 95% level of confidence. The hazard ratio (HR) is a measure used in survival analysis to compare the risk of an event occurring at any given point in time between two groups. Nomograms were generated and validated using R Statistical Software with the utilization of packages such as rms, survival, ggplot2, timeROC, and rmda. Statistical significance was determined when the *p*‐value was less than 0.05.

## 3 RESULTS

### 3.1 General characteristics

**FIGURE 1:**
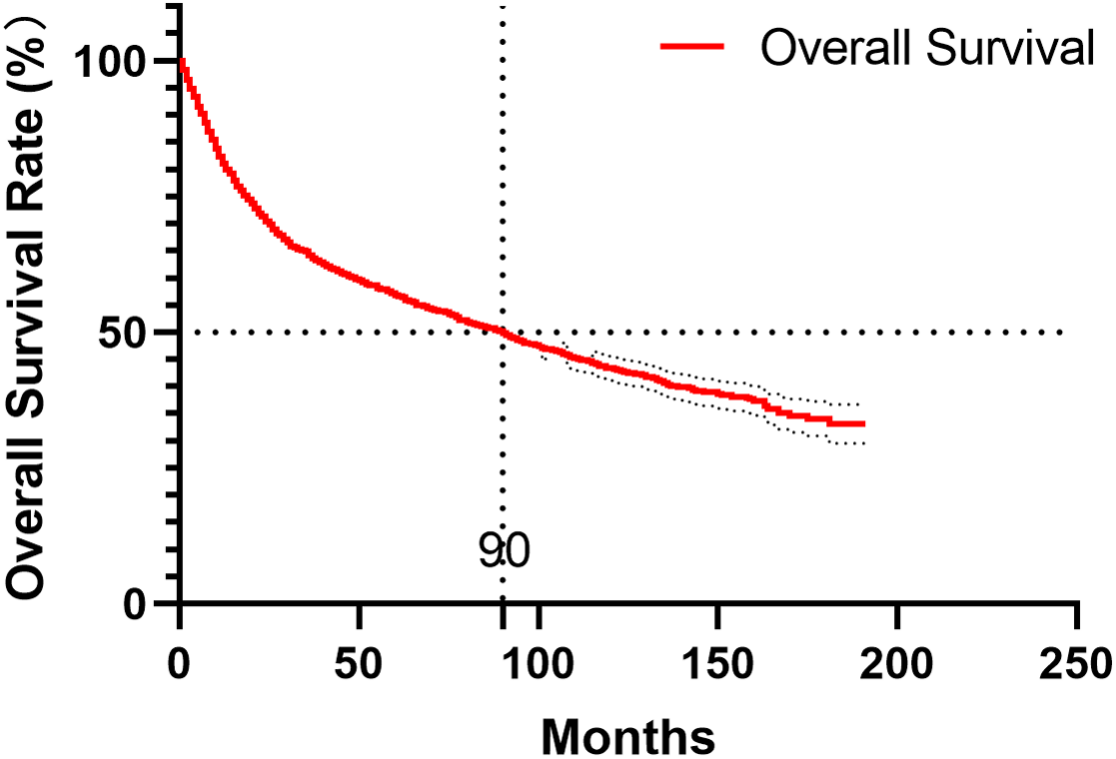
Kaplan–Meier survival curve for penile cancer patients.

A total of 5744 patients were retrieved from the SEER database, spanning from 2004 to 2019. Among these, 2246 patients were excluded as they did not meet the inclusion criteria, as depicted in Data S1. Consequently, 3498 patients were enrolled in this study, and they were randomly divided into two cohorts: the training group, consisting of 2450 patients, and the validation group 1, comprising 1048 patients. The baseline characteristics of the study population are presented in Table 1. Predominantly, the patients were Caucasian, with African American and individuals of other ethnicities representing 9.1% and 8.2%, respectively. A majority of the patients fell into Grade II, with 24.0%, 17.0%, and 0.7% in stages I, III, and IV, respectively. Squamous cell carcinoma emerged as the predominant histology. The patient distribution across stages was as follows: 1772 in stage T1, 738 in stage T2, 543 in stage T3, and 103 in stage T4. Over 91.7% of the patients underwent radiotherapy, while 87.1% received either partial or total surgery. Chemotherapy was administered to approximately 12.4% of the patients. The Kaplan–Meier survival curve for penile cancer patients is illustrated in Figure 1.

**TABLE 1:** Baseline characteristics of the study population.

	SEER		SYSMH	
	No		No	
Overall	3498		103	
Age, years (mean, SD)	64.68	(SD 14.80)	55.05	(SD 12.97)
Race				
White	2892	82.7%	0	0.0%
Black	320	9.1%	0	0.0%
Other	286	8.2%	103	100.0%
Primary site				
Unknown	1647	47.1%	13	12.6%
Prepuce	380	10.9%	16	15.5%
Glans	1121	32.0%	54	52.4%
Body	188	5.4%	11	10.7%
Overlapping lesion	162	4.6%	9	8.7%
Grade				
Unknown	710	20.3%	16	15.5%
Well (I)	839	24.0%	38	36.9%
Moderately (II)	1330	38.0%	44	42.7%
Poorly (III)	596	17.0%	5	4.9%
Undifferentiated (IV)	23	0.7%	0	0.0%
Histology				
Squamous cell carcinoma	3063	87.6%	90	87.4%
Verrucous carcinoma	191	5.4%	5	4.9%
Other/unknown	244	7.0%	8	7.8%
T stage				
T1	1772	50.7%	60	58.3%
T2	738	21.1%	27	26.2%
T3	543	15.5%	15	14.6%
T4	103	2.9%	0	0.0%
Ta	31	0.9%	1	1.0%
Tx	311	8.9%	0	0.0%
N stage				
N0	2425	69.3%	63	61.2%
N1	205	5.9%	18	17.5%
N2	243	6.9%	13	12.6%
N3	238	6.8%	8	7.8%
Nx	387	11.1%	1	1.0%
M stage				
M0	3193	91.3%	99	96.1%
M1	142	4.1%	3	2.9%
Mx	163	4.6%	1	1.0%
Surgery				
No/unknown	450	12.9%	3	2.9%
Partial surgery	2565	73.3%	75	72.8%
Total/Radical surgery	483	13.8%	25	24.3%
Non‐primary site surgery				
No	3408	97.4%	100	97.1%
Yes	90	2.6%	3	2.9%
Radiation				
No/unknown	3207	91.7%	97	94.2%
Yes	291	8.3%	6	5.8%
Chemotherapy				
No/unknown	3064	87.6%	91	88.3%
Yes	434	12.4%	12	11.7%
L/V invasion				
No	3124	89.3%	95	92.2%
Yes	374	10.7%	8	7.8%
Marital				
Married	1854	53.0%	100	97.1%
Other/unknown	1644	47.0%	3	2.9%
Income				
<$55,000	1045	29.9%	‐	‐
>$55,000	2453	70.1%	‐	‐
Rural–Urban				
Nonmetropolitan	273	7.8%	36	35.0%
Metropolitan	2904	83.0%	45	43.7%
Adjacent to metropolitan	321	9.2%	22	21.4%

Abbreviations: L/V Invasion, lymphangion/vessel invasion; SD, standard deviation; SEER, Surveillance, Epidemiology, and End Results.

### 3.2 Risk prediction nomogram development

**FIGURE 2:**
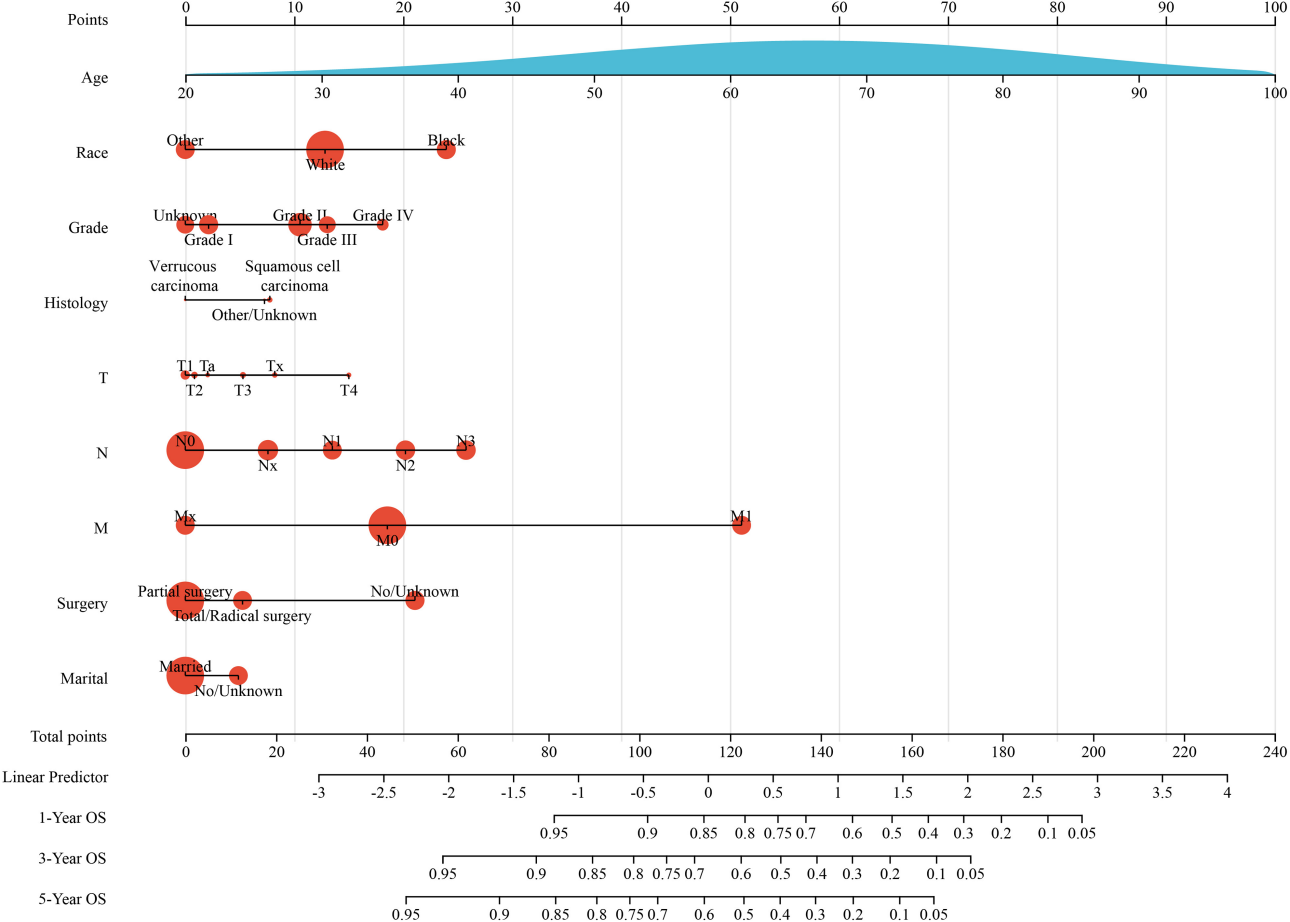
Nomogram predicting 1‐, 3‐, and 5‐year overall survival in penile cancer.

Both univariate and multivariate Cox regression analyses were conducted, with the findings detailed in Table 2. Stepwise regression method using the likelihood ratio (LR) test to identify the final independent risk factors in a multivariate analysis. Factors such as age, ethnicity, marital status, grade, histology, TNM stage, and surgery were recognized as independent prognostic indicators. Following this, a predictive model was developed based on these factors for both training groups. This model facilitates the projection of 1‐, 3‐, and 5‐year OS rates for patients, as demonstrated in the nomogram (Figure 2).

**TABLE 2:** Univariate and multivariate Cox analyses of patients with carcinoma of penis.

Variable	Univariate analysis			Multivariate analysis		
	HR	95% CI	*p*‐Value	HR	95% CI	*p*‐Value
Age, years	1.039	1.035–1.043	<0.001	–	–	–
Race	–	–	<0.001	–	–	<0.001
White	1 (ref)	–	–	1 (ref)	–	–
Black	1.273	1.083–1496	0.003	1.540	1.300–1.823	<0.001
Other	0.642	0.517–0.798	<0.001	0.639	0.511–0.799	<0.001
Primary site	–	–	0.032	–	–	–
Unknown	1 (ref)	–	–	–	–	–
Prepuce	0.777	0.652–0.925	0.005	–	–	–
Glans	0.961	0.858–1.076	0.491	–	–	–
Body	0.936	0.748–1.170	0.560	–	–	–
Overlapping lesion	1.137	0.898–1.440	0.286	–	–	–
Grade	–	–	<0.001	–	–	<0.001
Unknown	1 (ref)	–	–	1 (ref)	–	–
Well (I)	0.796	0.675–0.939	0.007	1.063	0.881–1.283	0.523
Moderately (II)	1.255	1.091–1.445	0.002	1.350	1.138–1.600	0.001
Poorly (III)	1.843	1.547–2.158	0.000	1.499	1.244–1.806	<0.001
Undifferentiated (IV)	1.782	1.060–2.996	0.029	1.323	0.722–2.265	<0.001
Histology	–	–	<0.001	–	–	0.012
Squamous cell carcinoma	1 (ref)	–	–	1 (ref)	–	–
Verrucous carcinoma	0.497	0.392–0.652	<0.001	0.660	0.492–0.885	0.006
Other/Unknown	0.930	0.760–1.137	0.478	1.016	0.795–1.298	0.901
T stage	–	–	<0.001	–	–	<0.001
T1	1 (ref)	–	–	1 (ref)	–	–
T2	1.364	1.198–1.554	<0.001	1.075	0.936–1.234	0.307
T3	1.945	1.701–2.225	<0.001	1.330	1.143–1.547	<0.001
T4	3.211	2.522–4.089	<0.001	2.046	1.559–2.684	<0.001
Ta	0.573	0.256–1.279	0.174	1.066	0.459–2.474	0.882
Tx	1.655	1.377–1.990	<0.001	1.211	0.940–1.561	0.139
N stage	–	–	<0.001	–	–	<0.001
N0	1 (ref)	–	–	1 (ref)	–	–
N1	2.039	1.692–2.458	<0.001	1.679	1.373–2.503	<0.001
N2	2.301	1.939–2.731	<0.001	1.917	1.584–2.321	<0.001
N3	3.127	2.648–3.693	<0.001	2.460	2.020–2.994	<0.001
Nx	1.386	1.170–1.642	<0.001	1.403	1.121–1.755	0.003
M stage	–	–	<0.001	–	–	<0.001
M0	1 (ref)	–	–	1 (ref)	–	–
M1	5.250	4.348–6.340	<0.001	2.661	2.142–3.307	<0.001
Mx	0.918	0.713–1.182	0.506	0.607	0.434–0.850	0.004
Surgery	–	–	<0.001	–	–	<0.001
No/Unknown	1 (ref)	–	–	1 (ref)	–	–
Partial surgery	0.411	0.359–0.470	<0.001	0.492	0.413–0.587	<0.001
Total/Radical surgery	0.671	0.565–0.796	<0.001	0.656	0.533–0.808	<0.001
Non‐primary site surgery	–	–	0.083	–	–	–
No	1 (ref)	–	–	–	–	–
Yes	1.304	0.978–1.738	0.071	–	–	–
Radiation	–	–	<0.001	–	–	–
No/Unknown	1 (ref)	–	–	–	–	–
Yes	1.727	1.476–2.020	<0.001	–	–	–
Chemotherapy	–	–	<0.001	–	–	–
No/Unknown	1 (ref)	–	–	–	–	–
Yes	1.807	1.579–2.067	<0.001	–	–	–
L/V Invasion	–	–	<0.001	–	–	–
No	1 (ref)	–	–	–	–	–
Yes	1.651	1.417–1.923	<0.001	–	–	–
Marital	–	–	<0.001	–	–	<0.001
Married	1 (ref)	–	–	1 (ref)	–	–
Other/Unknown	1.284	1.163–1.419	<0.001	1.126	1.015–1.250	0.025
Income	–	–	0.231	–	–	–
<$55,000	1 (ref)	–	–	–	–	–
>$55,000	0.936	0.841–1.042	0.504	–	–	–
Rural–Urban	–	–	0.093	–	–	–
Nonmetropolitan	1 (ref)	–	–	–	–	–
Metropolitan	0.887	0.742–1.060	0.186	–	–	–
Adjacent to metropolitan	1.036	0.824–1.302	0.765	–	–	–

Abbreviations: CI, confidence interval; HR, hazard ratio; L/V Invasion, lymphangion/vessel invasion.

### 3.3 Predictive accuracy of nomogram

**FIGURE 3:**
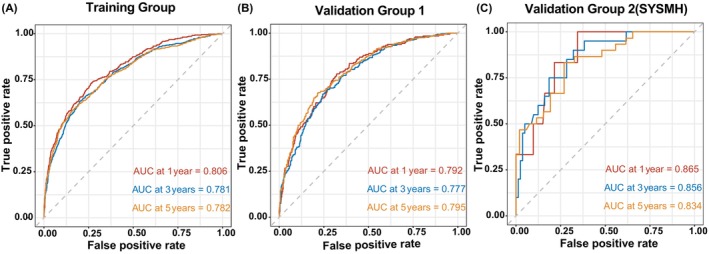
Receiver operating characteristic curve analysis. Comparing predictive accuracy in the training cohort (A), validation group 1 (B), and validation group 2 (C).

The precision of the nomogram in predicting 1‐, 3‐, and 5‐year OS was evaluated using the AUC index. For the training set, the AUC values were as follows: 0.806 at 1 year, 0.781 at 3 years, and 0.782 at 5 years, accompanied by a c‐index of 0.7424. Similarly, in validation group 1, the AUC values were 0.792 at 1 year, 0.777 at 3 years, and 0.795 at 5 years, with a c‐index of 0.7385. These results underscore the nomogram's robust discriminatory capacity, as shown in Figure 3A,B. Additionally, the calibration curves for both training and validation cohorts closely mirrored the ideal diagonal line, signifying exemplary model consistency. The calibration at 1 and 3 years is satisfactory, but the calibration at 5 years is less favorable for the SEER database. Furthermore, the calibration of the second validation database is inadequate. Data S2A–F display the nomogram‐predicted probabilities for 1‐, 3‐, and 5‐year OS. The decision curve analysis (DCA) results, illustrated in Data S3A–C, revealed a favorable net benefit for penile cancer patients using our model, underscoring its clinical applicability.

**FIGURE 4:**
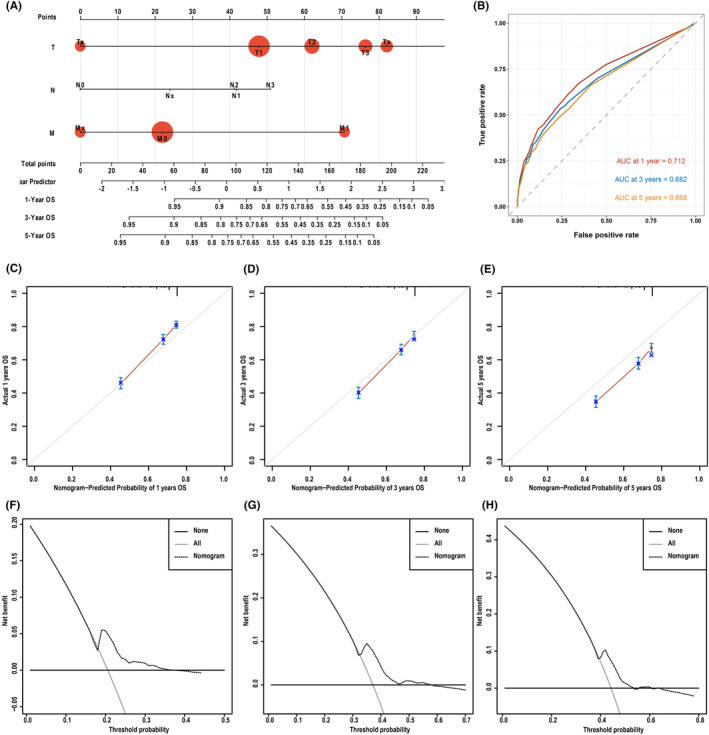
Comparative analysis and clinical utility of the nomogram. A nomogram for predicting 1‐, 3‐, and 5‐year overall survival (OS) based on the TNM stage (A), a comparison of ROC curves between the nomogram and TNM stage for predicting OS (B), calibration plots evaluating the nomogram's performance for 1‐, 3‐, and 5‐year OS predictions (C–E), and a DCA comparing the clinical utility of the OS prediction model over 1‐, 3‐, and 5‐year periods, juxtaposing the nomogram model and TNM stage model (F–H) with the range of threshold probabilities 0.201 (0.185, 0.229), 0.386 (0.374, 0.408), 0.442 (0.413, 0.461), respectively.

The nomogram was further validated externally with a cohort of 103 patients from SYSMH. The AUC values for this group at 1, 3, and 5 years were 0.865, 0.856, and 0.834, respectively, as depicted in Figure 4C, and the c‐index stood at 0.785, confirming the nomogram's high accuracy. This model also demonstrated strong consistency, as evidenced by the calibration curve for validation group 2 aligning closely with the ideal diagonal line (Data S2G–I). Additionally, the DCA showed significant net benefits for validation group 2 (Data S3G–I), highlighting the nomogram's considerable potential in aiding clinical decision‐making.

### 3.4 TNM based predictive nomogram construction

In addition, in the study, we constructed and validated a prediction model based on TNM staging, and compared the nomogram and TNM staging models to predict the survival of penile cancer (Figure 4A). The predicted AUC values of 1‐, 3‐, and 5‐year TMN stages were 0.712, 0.682, and 0.668, respectively, which were much lower than the nomogram we constructed (Figure 4B). Similarly, the comparison found that the nomogram constructed in this study had better ability to predict the prognosis of penile cancer due to its consistency with practice and clinical value (Figure 4C–H). These findings suggest that the nomogram may offer greater clinical utility compared to the TNM stage.

## 4 DISCUSSION

The prevalence of penile cancer is relatively low, as noted in sources,^9^, ^10^ but it exhibits substantial regional variation due to factors such as geographical location, religious practices, socioeconomic status, and general health conditions.^11^ Ethnic and religious groups, particularly Jewish and Muslim communities that traditionally practice early‐age circumcision, tend to have lower incidences of penile cancer.^12^ Treatment methods for the primary lesion include penile‐sparing treatments as well as radical penectomy with urethroperineal fistula or penile reconstruction. The choice of treatment method should be based on the size of the tumor, histological staging, grading, and the patient's own wishes. The overall principle is to remove the tumor completely while preserving as much of the penis as possible. However, the prognosis presents a significant challenge for medical professionals, primarily due to the rarity of cases and the absence of reliable prognostic tools.^13^ This scarcity has led to a lack of focused attention and guidelines for this patient demographic. Our study is pioneering in addressing this gap through the utilization of large‐scale data, including contributions from our own center.

The clinical stage of penile carcinoma is a critical determinant of prognosis. Post‐treatment survival rates show considerable variation by stage: 95.8% for stage I, 77.8% for stage II, 47.8% for stage III, and 0% for stage IV. Patients diagnosed with advanced penile cancer exhibit a 2‐year survival rate of only 21%.^3^ Lymph node metastasis, a key prognostic indicator, significantly impacts patient survival and necessitates a comprehensive assessment. While current treatments have boosted survival rates for about 80% of patients with early‐stage disease, those with inguinal lymph node metastases face drastically reduced 5‐year overall survival rates, dipping below 40%.^14^ Recent research underscores the relevance of inguinal lymph node density (LND) as a metric for risk stratification, particularly in penile cancer patients with positive lymph nodes who undergo inguinal lymph node dissection.^15^

The TNM stage system serves as a widely employed tool for evaluating cancer prognosis, but it is not the best tool for any one group. In this study, the constructed nomogram was compared with the predictive model based on the TNM stage, and it was found that our model has a higher value than the TNM in the aspect of prognosis assessment for penile cancer patients. Therefore, for clinical doctors, this tool is a necessary aid.^16^ In this study, we analyzed 3498 patients from the SEER database to explore the overall characteristics of penile carcinoma and to formulate prognostic prediction models. Through the application of Cox regression analysis, we discerned nine autonomous risk factors that include age, race, marital status, grade, histology, TNM stage, and surgical intervention, all serving as pivotal predictors of OS. Subsequently, we developed nomograms for 1‐, 3‐, and 5‐year OS. The validation of these nomograms focused on assessing their fit, generalizability, and effectiveness. The models showed comparatively good consistency, discriminatory capability, and clinical utility. Thus, these prediction models hold substantial potential in guiding clinical decisions for patients with penile carcinoma.

Yang et al.^17^ developed a competing risk prediction model using SEER database data, encompassing 2091 patients with penile cancer. Their findings indicated significant links between survival in penile cancer patients and factors such as AJCC stages II and III, tumor diameter exceeding 5 cm, and TNM stages N1‐3 and M1. Several prognostic factors influencing penile cancer prognosis have been identified.^4^, ^18^, ^19^ In our nomograms, the TNM stage is characterized by a high‐risk score, underscoring its importance. Models based on SEER data have demonstrated enhanced predictive accuracy for OS in penile cancer. In recent studies on marital status,^20^, ^21^ males who are unmarried or divorced had an increased risk of invasive penile squamous cell carcinoma, which may indicate a connection between advanced‐stage cancer and marital status. Our research supports this finding by identifying marital status as an independent risk factor for penile cancer.

In our study, the median patient age was 65 years, consistent with the age ranges reported in previous studies: 69 years by Zini et al.^6^ and 61 years by Zheng et al.^22^ Consistent with earlier findings, our study suggests that older individuals often have worse prognoses, likely due to coexisting health conditions. Moreover, tumor grade emerged as a notable independent prognostic factor in our Cox regression analysis, with higher‐grade tumors correlating with poorer outcomes. The predominance of squamous cell carcinoma in our findings may account for the generally unfavorable prognosis of penile carcinoma. Additionally, T, N, and M stages were identified as independent risk factors affecting OS. Our results reinforce the notion that surgical treatment can enhance patient outcomes, with local recurrences typically responding well to surgical interventions and exerting minimal impact on survival.^5^, ^14^, ^22^ In contrast, Chen et al.^13^ did not consider average income, which might have influenced their findings. However, our analysis revealed no significant effect of income or rural–urban status on OS. We attribute the robustness and reliability of our results to our larger sample size.

Nevertheless, our research does have certain limitations. The retrospective nature of our data collection constrained the breadth of our study. While circumcision may offer some protection against penile cancer, there is only limited information available on this topic. Hence, the generalizability of our nomogram to all penile cancer patients remains uncertain. Furthermore, we were unable to include potential prognostic factors such as smoking history, sexual behavior, and HPV infection^23^, ^24^, ^25^ in our analysis, which could have influenced our findings. Additionally, our approach mirrored previous studies in that we did not engage in prospective data collection. Therefore, validating the efficacy of our predictive model through large‐scale, prospective, randomized controlled studies is an essential next step.

## 5 CONCLUSION

In summary, our research, which used data from our institution and the SEER database, developed and externally validated a nomogram that can accurately predict the prognosis of patients with penile cancer. It indicated better consistency, a higher c‐index, and significant clinical value when compared to the TNM staging system's predictive model. This nomogram is a valuable tool that can help clinicians diagnose and treat penile cancer patients on an individual basis.

## AUTHOR CONTRIBUTIONS

**Jiawen Luo:** Writing – original draft (equal). **Jintao Hu:** Writing – review and editing (equal). **Yelisudan Mulati:** Writing – review and editing (equal). **Zhikai Wu:** Data curation (lead). **Cong Lai:** Methodology (lead). **Degeng Kong:** Data curation (supporting). **Cheng Liu:** Supervision (lead). **Kewei Xu:** Funding acquisition (lead).

## FUNDING INFORMATION

This work was funded by grants from the National Natural Science Foundation of China (Grant numbers: 82072841), Natural Science Foundation of Guangdong Province (Grant numbers: 2021A1515010199), Key Areas Research and Development Program of Guangdong (Grant numbers: 2020B111114002), Guangzhou Science and Technology Planning Project (Grant numbers: 202011020004 and 201803010029), Medical Science and Technology Research Foundation of Guangdong Province (Grant numbers: A2022541), Medical Scientific Research Foundation of Guangdong Province (Grant numbers: C2018060), Guangdong Provincial Clinical Research Center for Urological Diseases (Grant numbers: 2020B1111170006), Guangdong Science and Technology Department (Grant numbers: 2020B1212060018), Yixian Clinical Research Project of Sun Yat‐sen Memorial Hospital (Grant numbers: sys‐c‐201802), and Guangdong Province key areas research and development plan (2023B1111030006).

## CONFLICT OF INTEREST STATEMENT

The authors declare no conflicts of interest.

## ETHICS STATEMENT

This study was approved by the Research Ethics Committee of the Sun Yat‐sen Memorial Hospital. Before enrollment, each participant signed an informed consent form.

## CONSENT FOR PUBLICATION

Not applicable.

## Supporting information


Data S1.

Data S2.

Data S3.

## Data Availability

The original contributions presented in the study are publicly available. This data can be found here: https://seer.cancer.gov/.
