# Supplementary figures and images for: Developing and validating a nomogram for penile cancer survival: A comprehensive study based on SEER and Chinese data
Source: Cancer Med. 2024 Apr 3;13(7):e7111. doi: 10.1002/cam4.7111 (PMC10988236; doi:10.1002/cam4.7111)

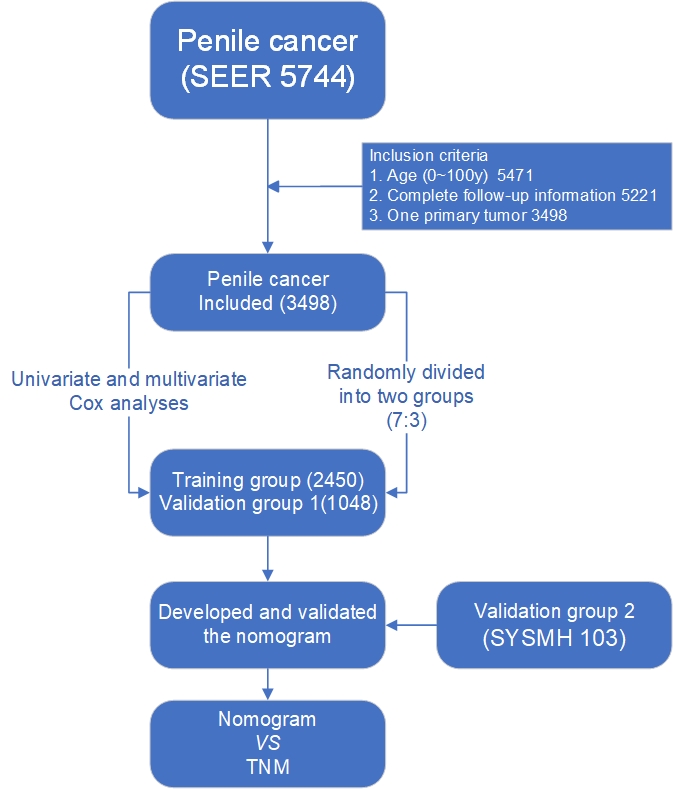

Supplement: Supplementary file 1 — Data S1. [file CAM4-13-e7111-s001.jpg]

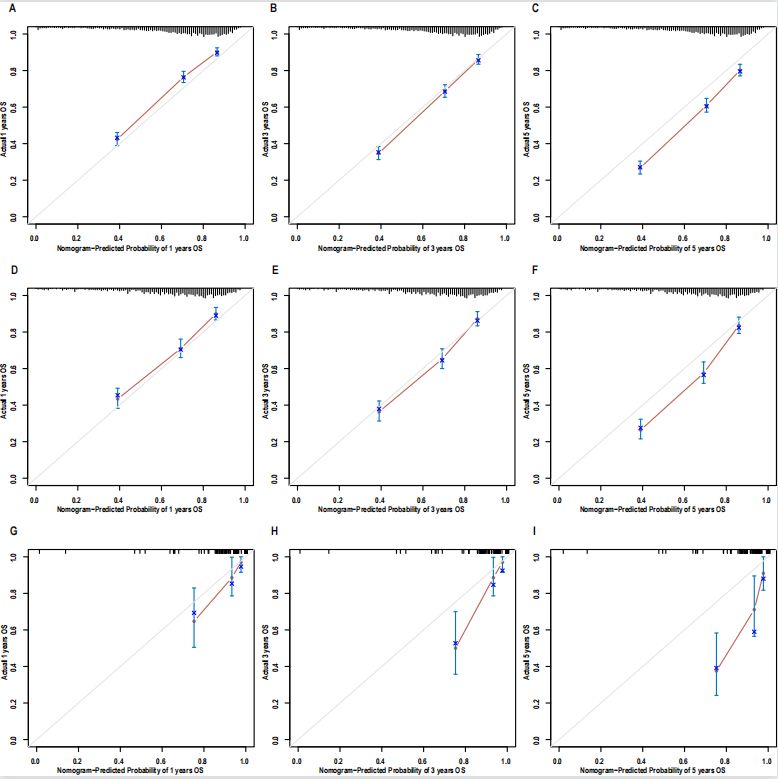

Supplement: Supplementary file 2 — Data S2. [file CAM4-13-e7111-s002.jpg]

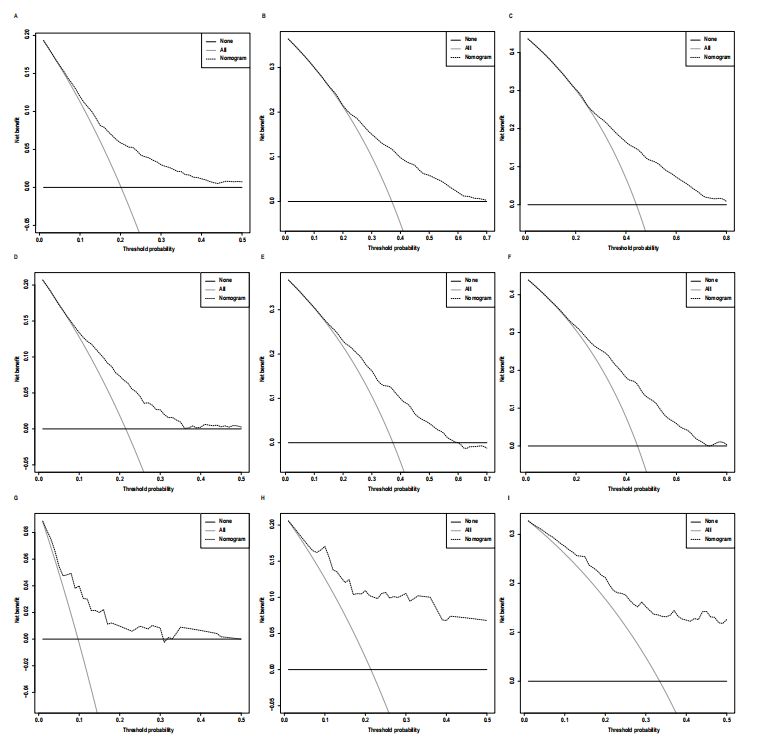

Supplement: Supplementary file 3 — Data S3. [file CAM4-13-e7111-s003.jpg]
